# Response to Parry et al.: Strong evidence for genomic integration of SARS-CoV-2 sequences and expression in patient tissues

**DOI:** 10.1073/pnas.2109497118

**Published:** 2021-08-03

**Authors:** Liguo Zhang, Alexsia Richards, M. Inmaculada Barrasa, Stephen H. Hughes, Richard A. Young, Rudolf Jaenisch

**Affiliations:** ^a^Whitehead Institute for Biomedical Research, Cambridge, MA 02142;; ^b^HIV Dynamics and Replication Program, Center for Cancer Research, National Cancer Institute, Frederick, MD 21702;; ^c^Department of Biology, Massachusetts Institute of Technology, Cambridge, MA 02142

Our paper (1) draws two conclusions:

1) SARS-CoV-2 sequences can integrate into the genome of infected cells that either overexpress (tables 1 and 2 of ref. 1) or do not overexpress (figure 2F of ref. 1) LINE1 by a LINE1-mediated retroposition mechanism.
2) We have identified large fractions of chimeric RNAs derived from negative-strand viral RNA in patients. Chimeric sequences containing negative-strand viral RNA are unlikely caused by artifacts associated with sequencing technology and likely derive from severe acute respiratory syndrome coronavirus 2 (SARS-CoV-2) sequences integrated into genomic DNA.

We summarize our responses to Parry et al. (2).

1) Parry et al. state that “SARS-CoV-2 integration into the host genome is unlikely.” Our response is that the “percent of library” calculation is not an estimate of integration frequency, which requires consideration of whole-genome sequencing (WGS) coverage. We identified integration events and observed two to five integrations per 10,000 cells at the current sequencing depth (Table 1). This is similar to the estimated integration frequency of lymphocytic choriomeningitis virus after acute infection (3). “Low frequency” of integration events cannot be interpreted as “unlikely.”
2) Parry et al. state that, because “‘insertions’ are found preferentially in protein-coding exons, a bias unknown to L1 endonuclease insertions . . ., these findings are likely spurious.” Our response is that we are not measuring L1 integrations but L1-mediated retroposition of SARS-CoV-2 RNA. Our data suggest that L1-mediated retroposition of RNA may have a different integration preference than L1.
3) Parry et al. state that “2 of the identified 61 chimeric nanopore genomic DNA (gDNA) reads contains human DNA from separate chromosomes.” Our response is that it is well known that nanopore sequencing occasionally ligates fragments from different chromosomes. Our demonstration of LINE1-mediated retroposition is based on sequence features of the integrated DNA and is not affected by these rare artifacts.
4) Parry et al. state that “larger pools of negative-sense SARS-CoV-2 chimeric reads may be due to differences in RNA extraction, ….” Our response is that there are no “larger pools of negative-sense chimeric reads.” The fraction of negative-strand RNAs produced during virus replication is 1,000- to 10,000-fold lower than that of positive-strand RNAs (figure 3 C and D in ref. 1). The low abundance of negative-strand RNA makes artifactual template fusion unlikely. The fraction of negative-strand viral RNAs detected in some patient tissues, which show no evidence of virus replication, are orders of magnitude higher than in cells with replicating virus (figure 3 E−G in ref. 1).
5) Parry et al. state that “there is no evidence of coronaviruses ever having integrated into the germline of host species.” Our response is that the lack of prior evidence is not an argument against new evidence. Whether coronavirus sequences are found in the germline of different species is irrelevant, as our experiments have focused on SARS-CoV2 integration into the genome of somatic cells and not into the germline.

**Table 1.** Summary of gDNA sequencing

Accession	Cell line	Strategy	Chimeric sequences	Genome-coverage (WGS)	Number of cells sequenced	Observed integrants per 10,000 cells
SRR14289057	HEK293T	Enrichment and Illumina	2		10,000	2
SRR14216062	Calu3	Enrichment and Illumina	3		10,000	3
SRR14216061	Calu3	Enrichment and Illumina	2		10,000	2
SRR14163829	HEK293T-L1	Nanopore WGS	63	18	160,000	4
SRR14136237	HEK293T-L1	Illumina WGS	8	10	50,000	2
SRR14136236	HEK293T-L1	Illumina WGS	9	16	20,000	5

## References

[1] L. Zhang., Reverse-transcribed SARS-CoV-2 RNA can integrate into the genome of cultured human cells and can be expressed in patient-derived tissues. Proc. Natl. Acad. Sci. U.S.A. 118, e2105968118 (2021). 33958444 10.1073/pnas.2105968118 PMC8166107

[2] R. Parry, R. J. Gifford, S. Lytras, S. C. Ray, L. Coin, No evidence of SARS-CoV-2 reverse transcription and integration as the origin of chimeric transcripts in patient tissues. Proc. Natl. Acad. Sci. U.S.A. 118, 10.1073/pnas.2109066118 (2021). PMC8379926 34344759

[3] P. Klenerman, H. Hengartner, R. M. Zinkernagel, A non-retroviral RNA virus persists in DNA form. Nature 390, 298–301 (1997). 9384383 10.1038/36876

